# Supplementary material for: Current Practices and Opportunities for Outpatient Parenteral Antimicrobial Therapy in Hospitals: A National Cross-Sectional Survey
Source: Antibiotics (Basel). 2022 Oct 1;11(10):1343. doi: 10.3390/antibiotics11101343 (PMC9598700; doi:10.3390/antibiotics11101343)
Supplement: Supplementary file 1 [file antibiotics-11-01343-s001.zip › antibiotics-1933538-supplementary.pdf]

Supplementary material

Index

- S1: Description of Dutch hospital organisation and antimicrobial stewardship teams
- S2: Survey development
- S3: PubMed literature search to identify surveys on current practices of OPAT
- S4: National survey Outpatient Parenteral Antimicrobial Therapy (OPAT)
- S5: OPAT patients yearly per 100 hospital beds
- S6: Barriers experienced by hospitals that do not perform OPAT

S1: Description of Dutch hospital organisation and AMS teams

In the Netherlands, only acute care hospitals provide OPAT care. Three types of acute care hospitals are distinguished, namely university hospitals, non-university teaching hospitals and non-teaching hospitals. Together, they provide care to all Dutch residents, with geographical location and sometimes specific expertise present determining where patients receive care. Since 2014, Dutch hospitals have been required to have an antimicrobial stewardship team that is responsible for the institutional ASP [1]. To date, all acute care hospitals have established such a program, which increasingly encompasses OPAT care [2]. Home-based infusion is the predominant OPAT care delivery model. Some patients discharged from the hospital are treated in nursing facilities. Infusion centres do not exist. In 2018, a Dutch practice guide on OPAT care was developed by the Dutch Working Party on Antibiotic Policy (SWAB) to support hospitals with shaping and implementing an OPAT program in their hospital [3].

S2: Survey development

First, we defined distinctive domains within OPAT care (e.g., patient selection and monitoring during OPAT) based on recent international clinical practice recommendations [4, 5] and a systematically developed set of 33 quality indicators (QIs) describing recommended OPAT care [6]. From these sources, we then identified recommendations about good OPAT care belonging to one of these domains.

Second, we prioritised these topics to limit the length of the survey and consequently stimulate a high response rate. Prioritisation was based on relevance and applicability to the Dutch setting and was guided by the Dutch practice guide on OPAT care [3].

Third, we compared our topics to previously published survey studies to identify previously overlooked, but relevant topics, see web-only Supplementary Material S3. Additionally, we used these studies to set up a list of potential barriers to OPAT care.

Fourth, we converted the identified topics into questions and added general questions on hospital characteristics and the ASP, see web-only Supplementary Material S4.

S3: Development of survey: PubMed literature search for surveys on current practices of OPAT

Search string

(((((((("Anti-Bacterial Agents"[Mesh] OR "Anti-Bacterial Agents" [Pharmacological Action] OR "Antibiotic Prophylaxis"[Mesh]))) OR "Bacterial Infections"[Mesh])) AND ((("Administration, Intravenous"[Mesh]) OR ("Infusions, Intravenous"[Mesh] OR "Administration, Intravenous"[Mesh] OR "Infusion Pumps"[Mesh])) OR "Infusions, Parenteral"[Mesh])) AND ("Outpatients"[Mesh] OR "Ambulatory Care"[Mesh] OR "Home Infusion Therapy"[Mesh] OR "Ambulatory Care Facilities"[Mesh]))) OR (((Outpatient*[tiab] OR out-patient*[tiab] OR home[tiab]) AND (parenteral[tiab] OR intravenous*[tiab] OR infusion[tiab]) AND (antibiotic*[tiab] OR antimicrobial[tiab] OR anti microbial[tiab] OR antifungal[tiab] OR anti fungal[tiab] OR antiparasitic*[tiab] OR anti parasitic*[tiab])) OR OPAT[tiab])

AND

(Survey [tiab] OR questionnaire [tiab] OR poll [tiab] OR inquiry [tiab])

Figure S1 Inclusion flowchart survey studies

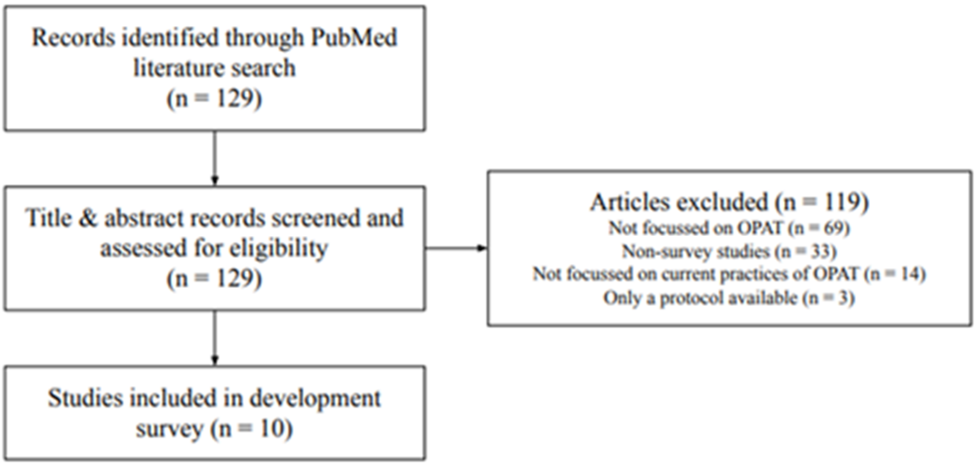

Ten survey studies were included in the development of our survey in the Netherlands [7-16].

S4: National survey Outpatient Parenteral Antimicrobial Therapy (OPAT)

On behalf of the SWAB working group on antimicrobial stewardship, we request you to participate in this annual survey. This year, the survey focuses on the current practices of OPAT in Dutch hospitals.

OPAT is an important part of antimicrobial stewardship, and the SWAB would like to inquire whether and how OPAT is applied and whether national support is necessary. The data collected with this survey will be published anonymously and aggregated.

We kindly request you to fill in a survey for every hospital location with an antimicrobial stewardship team. If one antimicrobial stewardship team works at several locations using the same methods, one survey is sufficient.

Participation will take around twenty minutes. During the process, you can close the survey and later continue where you left off. All answers will be treated confidentially. If any questions arise, please contact us, using the email-addresses below.

We kindly request you to return the survey before the 1st of December. Thank you for your participation!

On behalf of the Dutch Working Party on Antibiotic Policy (SWAB),
Jaap ten Oever, internist-infectious disease specialist SWAB Jaap.tenOever@radboudumc.nl
Hester Stoorvogel, PhD candidate internal medicine Hester.Stoorvogel@radboudumc.nl

Instructions:

- The survey consists of a maximum of 31 questions.
- For some questions (e.g. working hours), consultation with your colleagues of the antimicrobial stewardship team might be necessary
- Choose the answer that best fits your hospital. For most questions, one answer is possible. If not, it is indicated.
- All questions regarding ‘the hospital’ can be interpreted as regarding ‘all professionals that are involved with antimicrobial stewardship’.
- When any questions occur, do not hesitate to contact us:
  Hester Stoorvogel, PhD candidate internal medicine
  Hester.Stoorvogel@radboudumc.nl

General questions

1. Hereby, I give my consent to participate in this study and I consent that the data is collected and analysed for the purpose of this study.

O Yes

O No

2. I participate in this survey for the following hospital location(s)[i]:
   a. Hospital:…………………………..,
      location:………………………….

[i] We kindly request you to fill in a survey for every hospital location with an antimicrobial stewardship team. If one antimicrobial stewardship team works at several locations using the same methods, one survey is sufficient.

3. What is the total number of beds in the hospital?

   …… beds

4. What is the type of the hospital?

O University hospital

O Non-university teaching hospital

O Non-teaching hospital

OPAT team and organisation

5. Is Outpatient Parenteral Antimicrobial Therapy (OPAT) performed in your hospital?

O Yes

O No

6. How many patients are treated with OPAT annually?

….. patients annually

7. Did your hospital implement a team-based OPAT program[i]?

O Yes

O No

[i] A team-based OPAT program was defined as the presence of an OPAT team in the hospital whose roles and responsibility, as well as the OPAT care pathway, were defined.

8. Does this OPAT program formally fall under the responsibility of the antimicrobial stewardship team?

O Yes

O No

9. Which professions are part of the OPAT team? Multiple answers possible.

□ Pharmacist

□ Pharmacist assistant

□ Infectious disease specialist

□ Clinical microbiologist

□ Paediatrician

□ Transfer of care specialist

□ Nurse

□ Nurse specialist

□ Administrative assistant

□ IT specialist

□ Other: …..

10. How often does the OPAT team have policy meetings[i]?

…. times per year

[i] Meeting on functioning of the team, (future) policies, etc.

Patient selection for OPAT

11. Are inclusion and exclusion criteria established for the selection of patients for OPAT?

O Yes

O No

12. Is a consultation with an internist-infectious disease specialist mandatory prior to start of OPAT?

O Yes

O No

Antimicrobial treatment OPAT

13. Where does the administration of the first dose of antimicrobials take place? Multiple answers possible.

□ At home for outpatients

□ At the day care facility for outpatients

□ In the emergency department in patients who are not admitted but are discharged directly to their homes

□ In the ward where the patient was already admitted, before discharge

□ Other: …

14. Which intravenous access device is most frequently used for patients receiving OPAT for less than 7 days?

O Peripheral intravenous catheter

O Midline

O PICC

O Central vascular access device, other than PICC

O Other: …

15. Which intravenous access device is most frequently used for patients receiving OPAT for more than 7 days?

O Peripheral intravenous catheter

O Midline

O PICC

O Central vascular access device, other than PICC

O Other: …

16. Which infusion administration method is most frequently used in the hospital?[1]

O Electronic infusion pump

O Medication cassette reservoir

O Elastomeric pump

O Other: …

17. Are patients and caregivers offered the option of self-administration/administration by caregiver?

O Yes

O No

18. Are inclusion and exclusion criteria established for the selection of patients eligible for self-administration/administration by caregivers of OPAT?

O Yes

O No

19. What proportion of OPAT patients chooses self-administration or administration by caregivers of OPAT?

…..%

20. Is there a structured training program for patients and caregivers who choose to self-administer OPAT?

O Yes

O No

[1] *Electronic infusion pump* and *Medication cassette reservoir* were taken together as 'Electronic infusion pump' during the analysis because they both operate via an electronic infusion pump.

Monitoring during OPAT

21. Is the start of the treatment of OPAT reported in writing to the patient's general physician (or geriatrist)?

O Yes

O No

22. Medical responsibility for OPAT treatment may lie with the treating physician or the OPAT team. In what proportion of OPAT patients does the OPAT team also become medically responsible for OPAT treatment?

…..%

23. Is there a protocol for laboratory follow-up (excluding drug concentrations) during OPAT?

O Yes

O No

Results after OPAT

24. Which of the following outcome measures are measured and recorded for individual OPAT patients?

□ No outcome measures

□ Completion of OPAT as planned

□ Side effects of antimicrobials

□ Clinical outcome

□ Survival of patients during OPAT

□ Patient experiences

□ Emergency department visit during OPAT

□ Readmission

□ Venous access complication or infection

□ Other: …

Barriers to OPAT

25. Please state to what extent you do or do not agree with the following statements:

Our OPAT team / hospital is lacking…

a. *Financial support for OPAT*
Strongly disagree – disagree – slightly disagree – slightly agree – agree – strongly agree

b. *Mandate of the hospital board for OPAT*
Strongly disagree – disagree – slightly disagree – slightly agree – agree – strongly agree

c. *A team-based OPAT program*
Strongly disagree – disagree – slightly disagree – slightly agree – agree – strongly agree

d. *Leadership in the hospital relating to OPAT*
Strongly disagree – disagree – slightly disagree – slightly agree – agree – strongly agree

e. *Knowledge of health professionals about OPAT*
Strongly disagree – disagree – slightly disagree – slightly agree – agree – strongly agree

f. *A local protocol for OPAT*
Strongly disagree – disagree – slightly disagree – slightly agree – agree – strongly agree

g. *IT support for OPAT*
Strongly disagree – disagree – slightly disagree – slightly agree – agree – strongly agree

h. *Administrative support for OPAT*
Strongly disagree – disagree – slightly disagree – slightly agree – agree – strongly agree

i. *Time of health professionals for OPAT*
Strongly disagree – disagree – slightly disagree – slightly agree – agree – strongly agree

j. *Interest in OPAT of eligible patients*
Strongly disagree – disagree – slightly disagree – slightly agree – agree – strongly agree

k. *Communication between health professionals about OPAT*
Strongly disagree – disagree – slightly disagree – slightly agree – agree – strongly agree

l. *Logistics to get blood drawn and receive results quickly*
Strongly disagree – disagree – slightly disagree – slightly agree – agree – strongly agree

26. Are there other barriers you experience to perform OPAT?

O Yes, ……

O No

27. What makes your OPAT program a success?

THANK YOU FOR YOUR PARTICIPATION IN THIS SURVEY

S5: OPAT patients yearly per 100 hospital beds

<u>Figure S2 Scatter plot of the variance in OPAT patients yearly per 100 hospital beds for both hospitals with (n=22) and without (n=33) an OPAT program</u>

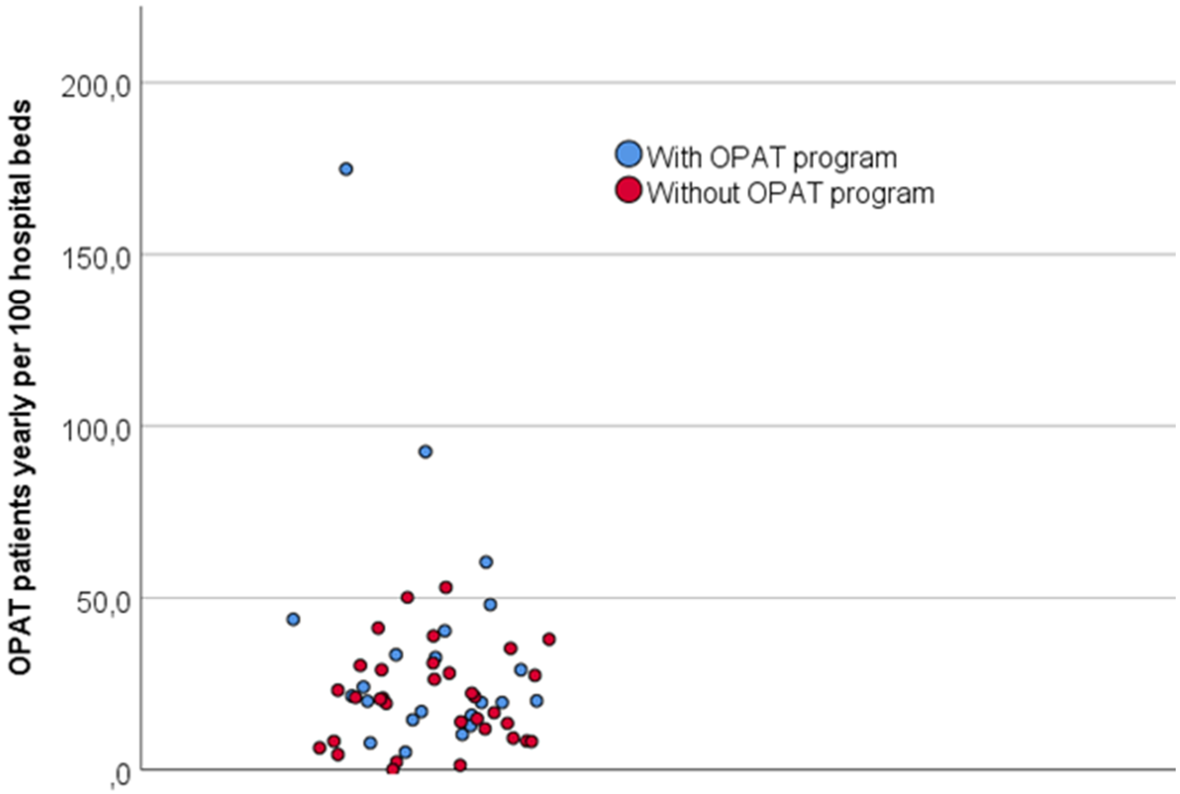

S6: Barriers experienced by hospitals that do not perform OPAT

Figure S3 Barriers experienced by hospitals that do not perform OPAT (n=5)[a]

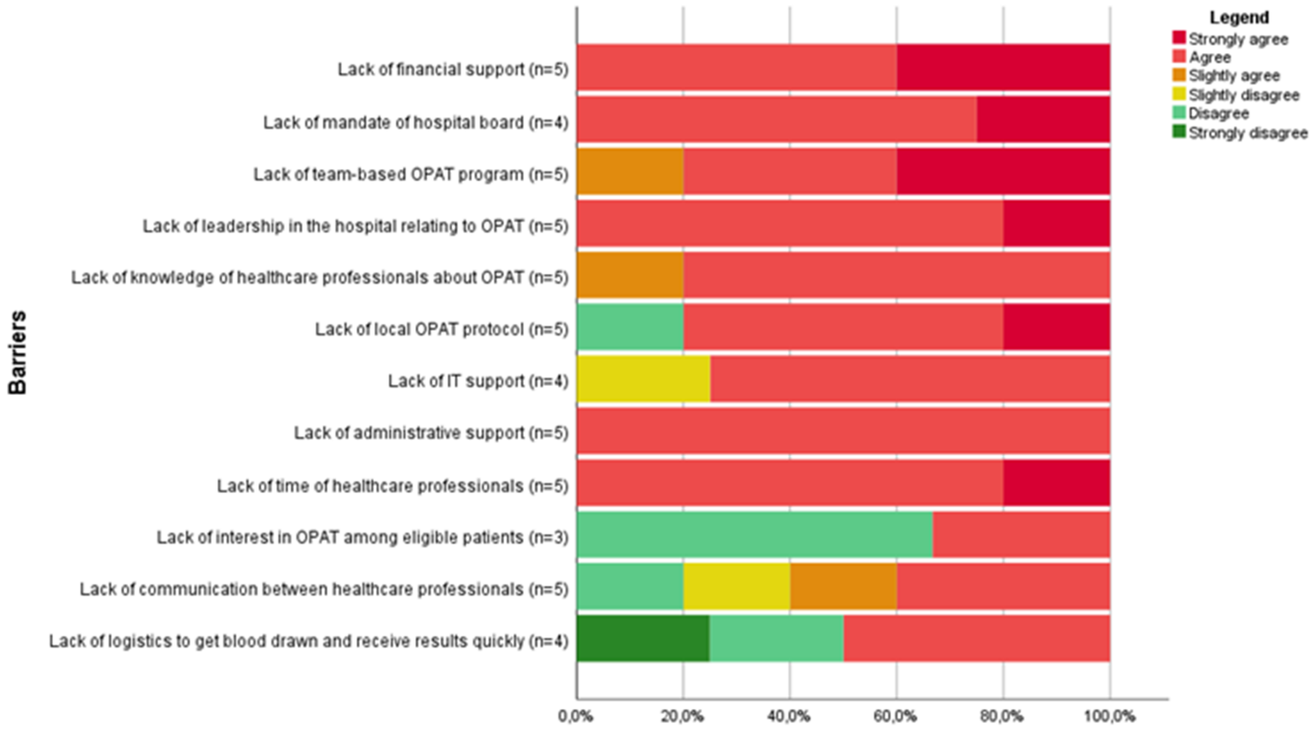

[a] The value 'Do not know' was considered as missing, resulting in different denominators per barrier.

Figure S4 Barriers experienced by hospitals with (n=22) and without (n=33) an OPAT program[a]

Barriers experienced by hospitals with an OPAT program (n=22)[a]:

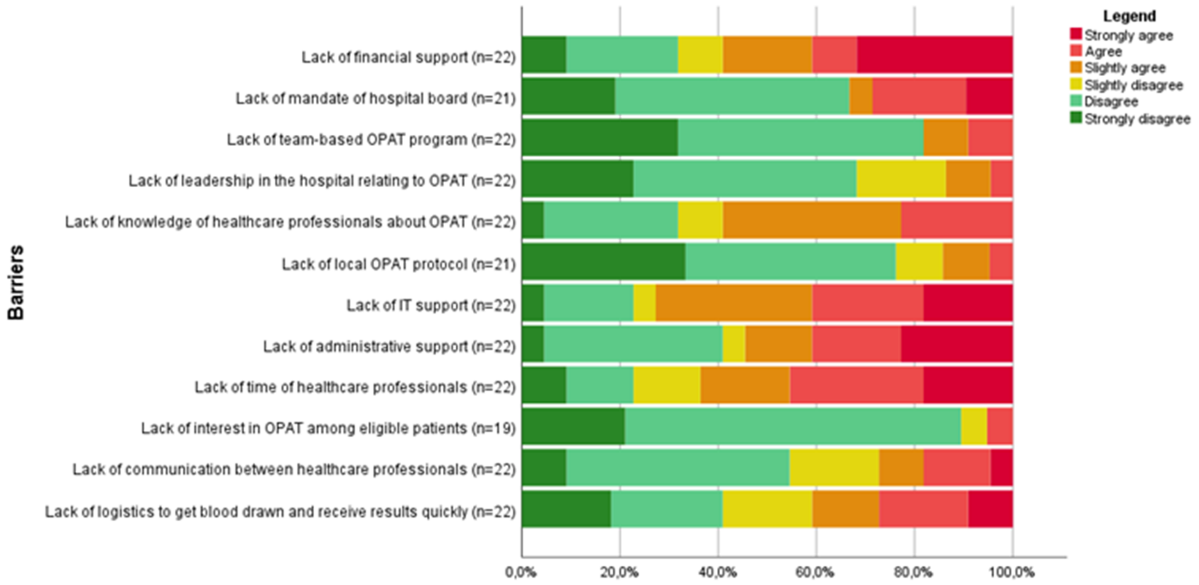

[a] The value 'Do not know' was considered as missing, resulting in different denominators per barrier.

Barriers experienced by hospitals without an OPAT program (n=33)[a]:

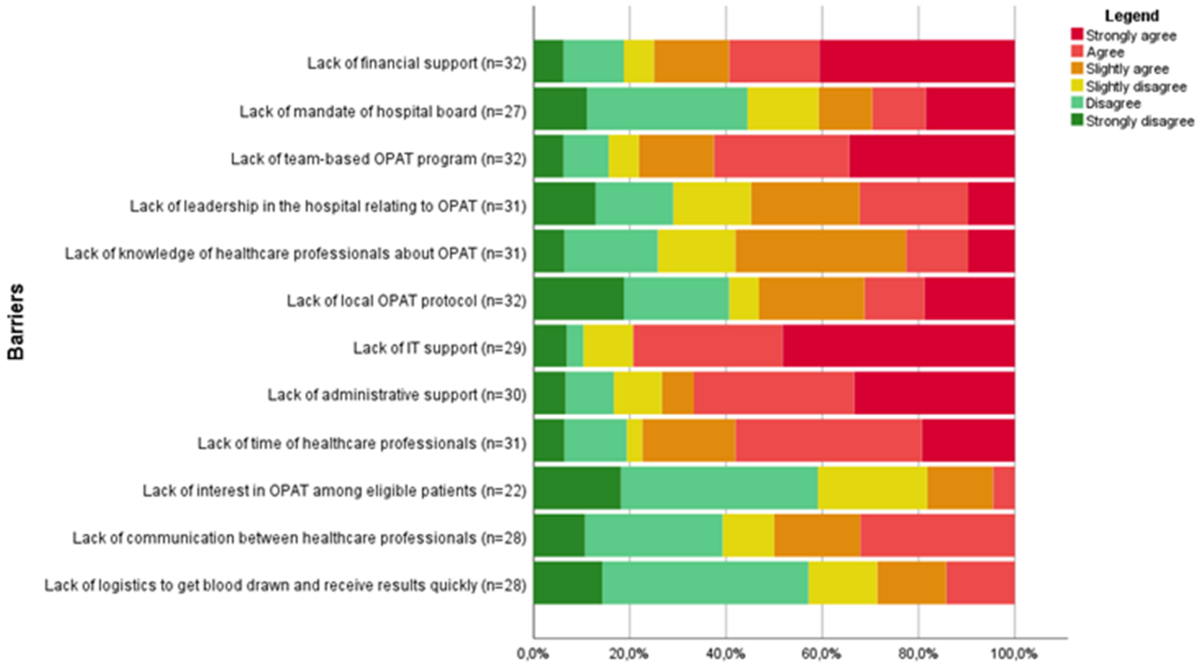

[a] The value 'Do not know' was considered as missing, resulting in different denominators per barrier.

References

1 Ten Oever J, Harmsen M, Schouten J, et al. Human resources required for antimicrobial stewardship teams: a Dutch consensus report. Clin Microbiol Infect. 2018;24(12):1273-9. DOI: 10.1016/j.cmi.2018.07.005.

2 Dutch Working Party on Antibiotic Policy (SWAB). NethMap 2021: Consumption of antimicrobial agents and antimicrobial resistance among medically important bacteria in the Netherlands; 2021.

3 Dutch Working Party on Antibiotic Policy (SWAB). Praktijkgids: Implementatie van een OPAT-programma in het ziekenhuis. N.d. Available from: https://swab.nl/en/opat-practice-guide.

4 Chapman ALN, Patel S, Horner C, et al. Updated good practice recommendations for outpatient parenteral antimicrobial therapy (OPAT) in adults and children in the UK. JAC Antimicrob Resist. 2019;1(2):dlz026. DOI: 10.1093/jacamr/dlz026.

5 Norris AH, Shrestha NK, Allison GM, et al. 2018 Infectious Diseases Society of America Clinical Practice Guideline for the Management of Outpatient Parenteral Antimicrobial Therapy. Clin Infect Dis. 2019;68(1):e1-e35. DOI: 10.1093/cid/ciy745.

6 Berrevoets MAH, Ten Oever J, Oerlemans AJM, et al. Quality Indicators for Appropriate Outpatient Parenteral Antimicrobial Therapy in Adults: A Systematic Review and RAND-modified Delphi Procedure. Clin Infect Dis. 2020;70(6):1075-82. DOI: 10.1093/cid/ciz362.

7 Banerjee R, Beekmann SE, Doby EH, et al. Outpatient Parenteral Antimicrobial Therapy Practices Among Pediatric Infectious Diseases Consultants: Results of an Emerging Infections Network Survey. J Pediatric Infect Dis Soc. 2014;3(1):85-8. DOI: 10.1093/jpids/pis137.

8 Chary A, Tice AD, Martinelli LP, et al. Experience of infectious diseases consultants with outpatient parenteral antimicrobial therapy: results of an emerging infections network survey. Clin Infect Dis. 2006;43(10):1290-5. DOI: 10.1086/508456.

9 Durojaiye OC, Cartwright K, Ntziora F. Outpatient parenteral antimicrobial therapy (OPAT) in the UK: a cross-sectional survey of acute hospital trusts and health boards. Diagn Microbiol Infect Dis. 2019;93(1):58-62. DOI: 10.1016/j.diagmicrobio.2018.07.013.

10 Fisher D, Michaels J, Hase R, et al. Outpatient parenteral antibiotic therapy (OPAT) in Asia: missing an opportunity. J Antimicrob Chemother. 2017;72(4):1221-6. DOI: 10.1093/jac/dkw551.

11 Hamad Y, Lane MA, Beekmann SE, et al. Perspectives of United States-based Infectious Diseases Physicians on Outpatient Parenteral Antimicrobial Therapy Practice. Open Forum Infect Dis. 2019;6(10). DOI: 10.1093/ofid/ofz363.

12 Lane MA, Marschall J, Beekmann SE, et al. Outpatient parenteral antimicrobial therapy practices among adult infectious disease physicians. Infect Control Hosp Epidemiol. 2014;35(7):839-44. DOI: 10.1086/676859.

13 Muldoon EG, Allison GM, Gallagher D, et al. Outpatient parenteral antimicrobial therapy (OPAT) in the Republic of Ireland: results of a national survey. Eur J Clin Microbiol Infect Dis. 2013;32(11):1465-70. DOI: 10.1007/s10096-013-1899-4.

14 Muldoon EG, Switkowski K, Tice A, et al. A national survey of infectious disease practitioners on their use of outpatient parenteral antimicrobial therapy (OPAT). Infect Dis (Lond). 2015;47(1):39-45. DOI: 10.3109/00365548.2014.967290.

15 Seaton RA, Nathwani D. Outpatient and home parenteral antibiotic therapy (OHPAT) in the UK: survey of infection specialists' experience and views. Clin Microbiol Infect. 2000;6(7):387-90.

16 Vaz LE, Felder KK, Newland JG, et al. A National Survey of Outpatient Parenteral Antibiotic Therapy Practices. J Pediatric Infect Dis Soc. 2022;11(3):115-8. DOI: 10.1093/jpids/piab127.
